# Supplementary material for: Opportunistic assessment of ischemic heart disease risk using abdominopelvic computed tomography and medical record data: a multimodal explainable artificial intelligence approach
Source: Sci Rep. 2023 Nov 29;13:21034. doi: 10.1038/s41598-023-47895-y (PMC10687235; doi:10.1038/s41598-023-47895-y)
Supplement: Supplementary file 1 — Supplementary Information. [file 41598_2023_47895_MOESM1_ESM.pdf]

Appendix: Supplemental Figures and Tables

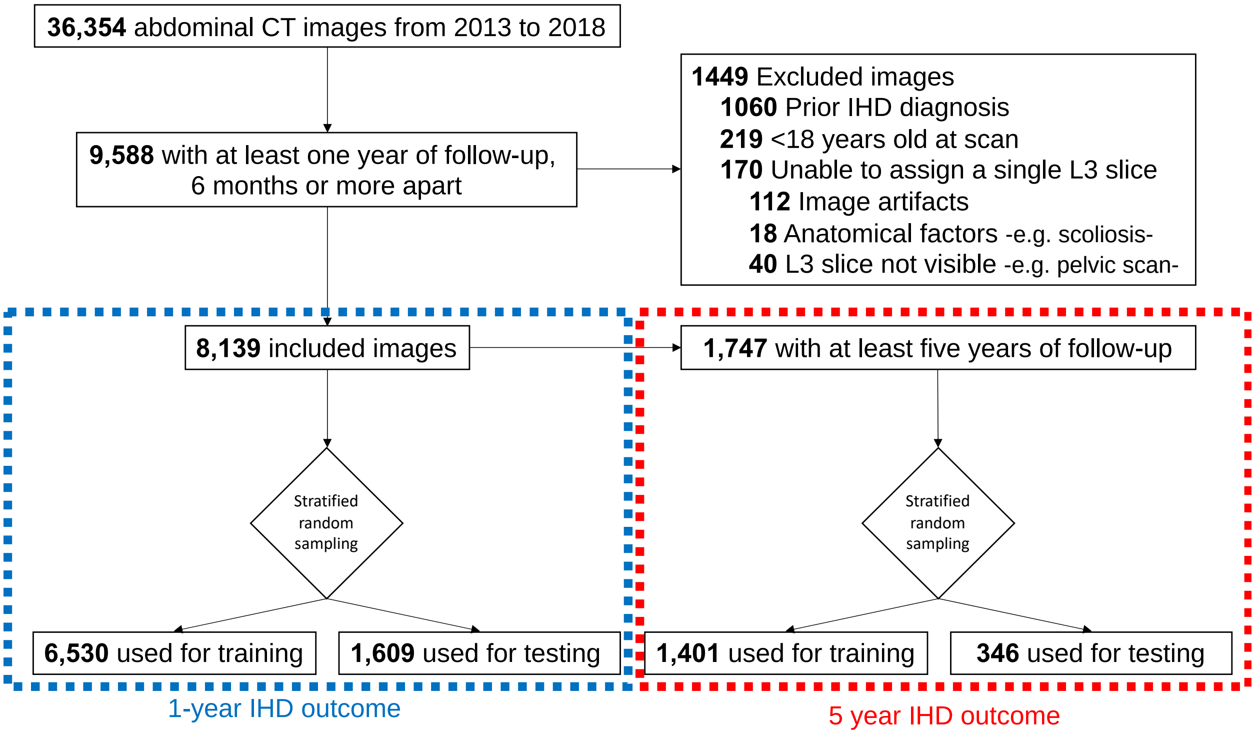

Supplemental Figure 1. Sampling criteria to arrive at final cohorts.

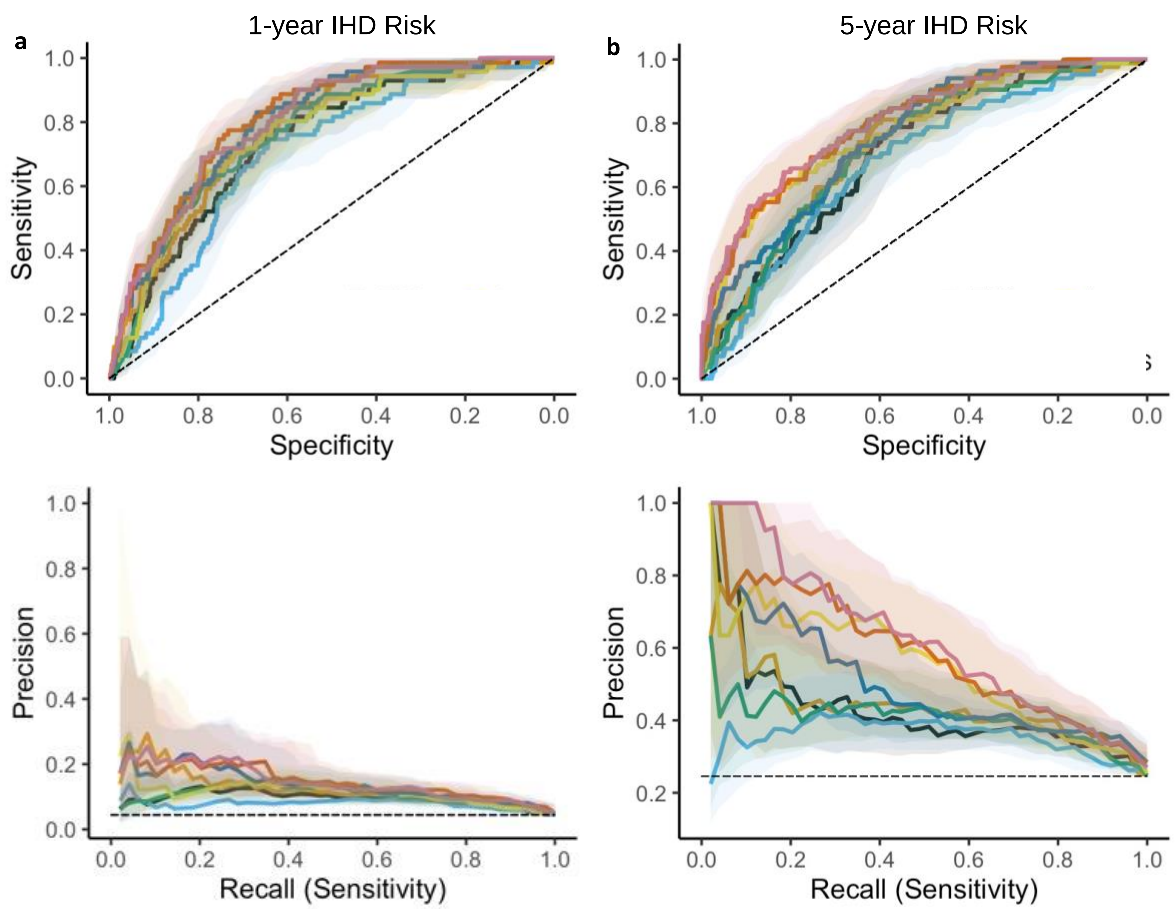

	1y AUROC (95% CI)	1y AUCPR (95% CI)	5y AUROC (95% CI)	5y AUCPR (95% CI)
FRS	.74 (.68-.79)	.10 (.08-.13)	.71 (.65-.77)	.43 (.36-.52)
PCE	.77 (.71-.81)	.13 (.10-.19)	.73 (.68-.79)	.44 (.38-.54)
Segmentation only (S)	.70 (.65-.76)	.08 (.07-.12)	.68 (.62-.75)	.36 (.31-.44)
PCE + Segmentation (PCE + S)	.76 (.71-.81)	.11 (.09-.14)	.72 (.66-.78)	.41 (.35-.51)
Imaging only (I)	.76 (.70-.81)	.12 (.09-.19)	.78 (.71-.83)	.56 (.47-.66)
Clinical Only (C)	.80 (.75-.84)	.14 (.11-.21)	.76 (.71-.81)	.51 (.43-.61)
Imaging + Clinical Fusion (I+C)	**.81 (.76-.85)**	**.15 (.12-.22)**	**.80 (.74-.85)**	**.60 (.51-.70)**
Imaging + Clinical + Segmentation Fusion (I+C+S)	.80 (.75-.84)	**.15 (.11-.22)**	**.81 (.75-.86)**	**.63 (.54-.72)**

1y AUROC *P*-value

Model	FRS	PCE	S	PCE+S	C	I	I+C	I+C+S
FRS	-	.07	.20	.17	**.004**	.41	**<.001**	**.02**
PCE	-	-	**.02**	.83	.08	.89	**.02**	.14
S	-	-	-	**.01**	**<.001**	**.04**	**<.001**	**.002**
PCE+S	-	-	-	-	**.04**	.98	**.006**	.09
C	-	-	-	-	-	.18	.19	.95
I	-	-	-	-	-	-	**.01**	.059
I+C	-	-	-	-	-	-	-	.16
I+C+S	-	-	-	-	-	-	-	-

5y AUROC *P*-value

Model	FRS	PCE	S	PCE+S	C	I	I+C	I+C+S
FRS	-	.14	.42	.58	**.047**	.08	**.007**	**.004**
PCE	-	-	.10	.40	.27	.25	**.03**	**.02**
S	-	-	-	.10	**.01**	**.005**	**<.001**	**<.001**
PCE+S	-	-	-	-	**.09**	**.11**	**.007**	**.005**
C	-	-	-	-	-	.58	.10	**.047**
I	-	-	-	-	-	-	**.006**	**.01**
I+C	-	-	-	-	-	-	-	.25
I+C+S	-	-	-	-	-	-	-	-

1y AUCPR *P*-value

Model	FRS	PCE	S	PCE+S	C	I	I+C	I+C+S
FRS	-	.05	.34	.30	.07	.29	**.03**	.06
PCE	-	-	**.04**	.38	.70	.76	.49	.53
S	-	-	-	.09	**.03**	.10	**.01**	**.03**
PCE+S	-	-	-	-	.15	.62	.08	.10
C	-	-	-	-	-	.50	.29	.42
I	-	-	-	-	-	-	.26	.28
I+C	-	-	-	-	-	-	-	.80
I+C+S	-	-	-	-	-	-	-	-

5y AUCPR *P*-value

Model	FRS	PCE	S	PCE+S	C	I	I+C	I+C+S
FRS	-	.79	.09	.61	.13	**.047**	**.005**	**<.001**
PCE	-	-	.06	.39	.14	**.048**	**.003**	**<.001**
S	-	-	-	.13	**.001**	**<.001**	**<.001**	**<.001**
PCE+S	-	-	-	-	**.02**	**.006**	**<.001**	**<.001**
C	-	-	-	-	-	.42	**.047**	**.002**
I	-	-	-	-	-	-	.07	**.007**
I+C	-	-	-	-	-	-	-	**.02**
I+C+S	-	-	-	-	-	-	-	-

Supplemental Figure 2. Proposed model performance in comparison to pooled cohort Equations (PCE) and Framingham risk score (FRS) as measured by receiver operating characteristic and precision-recall curves for 1-year (a) and 5-year (b) IHD risk prediction. Shaded areas correspond to 95% confidence intervals. In Figure legend area under receiver operating characteristic and precision-recall curves, the AUROC and AUCPR curves are shown. Below, *P* values for pair-wise comparisons between models are reported. Confidence intervals (CI) and *P* values were obtained using the DeLong method for AUROC and the bootstrap method for AUCPR.

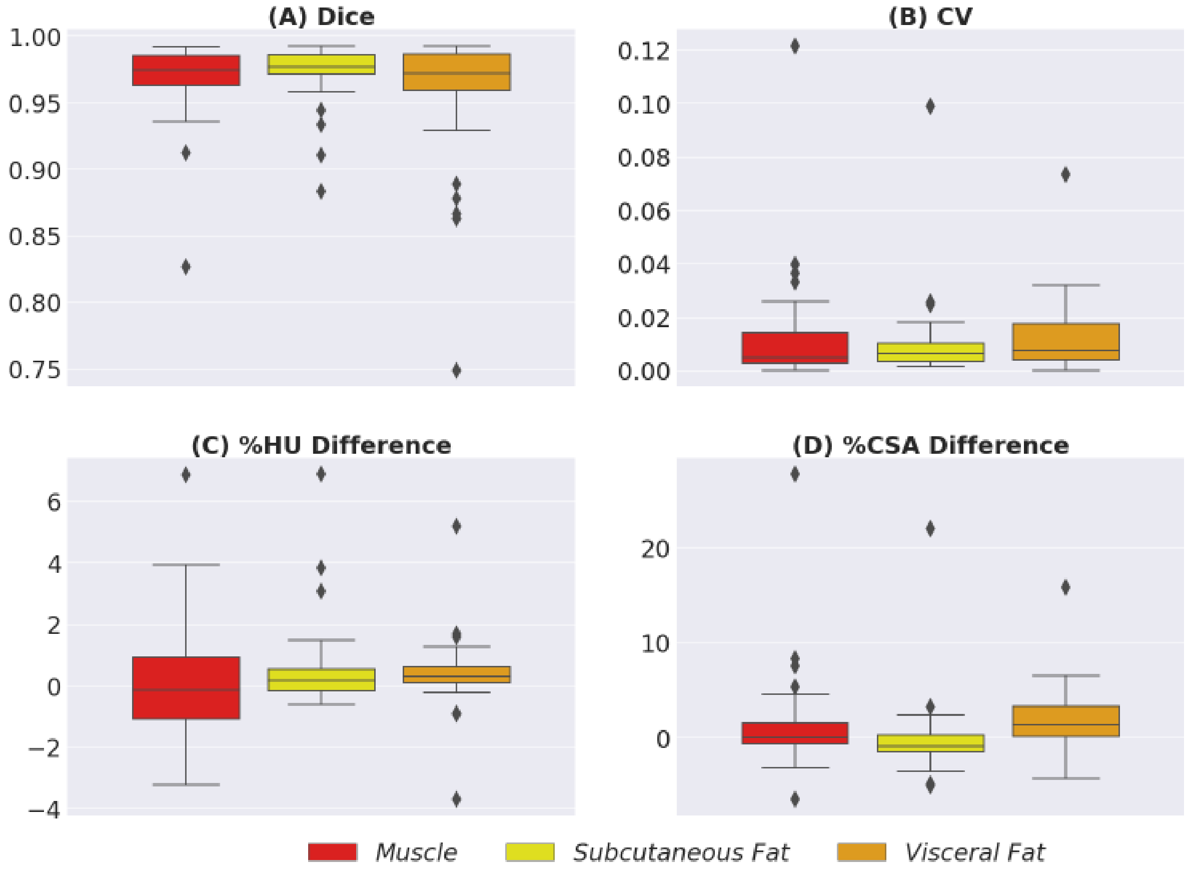

Supplemental Figure 3. Segmentation model performance. CV : coefficient of variation, HU: Hounsfield Unit, CSA: cross-sectional area. Center line represents median, box limits upper and lower quartiles, whiskers 1.5x interquartile range, and points outliers.

Supplemental Table 1. Validation performance of alternative training schemes and hyperparameters of predictive models. *,**: hyperparameters selected for 1-year and 5-year IHD prediction, respectively.

Model	Hyperparameters tested	Best Validation AUROC	
		1y	5y
**Segmentation only**	C: {.001, 0.1, 0.5, 1*,**,5,10}	.6771	.7038
**Imaging only**			
Selected configuration	Batch size 8<br>Learning rate {7e-6*,6e-6**}<br>Adam optimizer<br>Max epochs {10*,10**}<br>Cross Entropy Loss	.7733	.7947
Training only final layer	Batch size 8<br>Learning rate {1e-4,1e-5,1e-6}<br>Adam optimizer<br>Max epochs 15<br>Cross Entropy Loss	.5656	.6523
Image augmentations	Batch size 8<br>Learning rate {1e-3, 1e-4, 1e-5, 1e-6}<br>Adam optimizer<br>Max Epochs 20<br>Cross Entropy Loss<br>Rotations of up to 3° and pixel shifting of up to 5 pixels during training	.7089	.7867
Focal loss	Batch size 8<br>Learning rate {1e-4,1e-5, 1e-6}<br>Adam optimizer<br>Max epochs 20<br>Focal loss (gamma:{2,3,4,5,10} alpha:{[.01,.99],[0.05,0.95]})<br>Augmentations {Yes/No}	.7275	.7879
**Clinical only**	colsample_bytree:{.2,.3,.4,.5,.6,.7,.8,.9*,1**}<br>gamma:{.1,.5,1,5*,10**}<br>max_depth: {1,2,3,4*,**,5,6}<br>min_child_weight: {.1,.5,1*,**}<br>subsample: {.5*,.6,.7,.8, .9*,1**}<br>learning_rate: {.001,.01,.05*,.1**,.3, 1}<br>scale_pos_weight:{1*,**,5,10,15,20}	.7778	.7820
**PCE + segmentation**	colsample_bytree:{.2,.4,.6,.8**,1*}<br>gamma:{.1,.5,1,10*,**}<br>max_depth: {1,2**,3*,4,5,6}<br>min_child_weight: {.1**,.5*,1}<br>subsample: {.5**, .75, 1*}	.7421	.7366
**Imaging + Clinical Fusion**	C: {0.1**,0.5,1,5,10,20,30, 50, 100*, 500, 1000}	.9434	.9215
**Imaging + Clinical + Segmentation Fusion**	C: {0.1,0.5,1,5,10,20,30, 50, 100*,**, 500, 1000}	.9466	.9241

Supplemental Table 2. Variables captured from the electronic medical record and used as covariates of Clinical only model. (See attached file).

Supplemental Table 3. Distribution of demographic variables across the study population. Features relevant to pooled cohort equations risk assessment, as well as body composition metrics derived from segmentation are also included.

Variable	1-year follow-up								5-year follow-up							
	Train				Test				Train				Test			
	non-IHD (n=6246)		IHD (n=284)		non-IHD (n=1538)		IHD (n=71)		non-IHD (n=1046)		IHD (n=355)		non-IHD (n=261)		IHD (n=85)	
	% / Mean (SD)	missing	% / Mean (SD)	missing	% / Mean (SD)	missing	% / Mean (SD)	missing	% / Mean (SD)	missing	% / Mean (SD)	missing	% / Mean (SD)	missing	% / Mean (SD)	missing
Age	51.0 (17.1)	0	63.7 (14.1)	0	51.3 (16.7)	0	66.1 (16.2)	0	51.5 (16.4)	0	63.3 (15.1)	0	51.0 (16.9)	0	64.6 (14.7)	0
Male Sex	39.7	0	50.4	0	40.9	0	54.9	0	36.7	0	51.5	0	35.6	0	41.2	0
Smoking	6.2	184	3.9	1	6.9	50	11.6	2	5.6	30	6.3	3	5.6	9	7.1	0
Race/Ethnicity		0		0		0		0		0		0		0		0
White	47.7		45.1		50.3		45.1		53.1		44.8		52.5		48.2	
Black	4.1		2.8		3.1		2.8		2.7		2.8		5.4		3.5	
Hispanic	21.2		20.4		20.3		22.5		18.4		21.1		19.5		16.5	
Asian	16.3		20.1		15.7		18.3		15.0		20.0		14.5		19.4	
Other/Unknown	10.6		11.6		10.5		11.3		10.9		11.3		9.6		12.9	
Treated for hypertension	25.0	0	50.4	0	24.8	0	49.3	0	28.0	0	50.4	0	27.2	0	51.8	0
Diabetes diagnosis	13.4	0	26.1	0	14.7	0	25.4	0	13.4	0	25.6	0	9.2	0	28.2	0
HDL Cholesterol [mg/dL]	56.6 (20.4)	5100	58.4 (22.1)	284	57.1 (19.7)	1217	52.3 (16.5)	58	57.8 (23.3)	833	56.3 (19.6)	270	51.9 (18.0)	211	59.6 (25.0)	64
Total Cholesterol [mg/dL]	184.3 (47.3)	5037	175.1 (50.5)	216	183.9 (42.2)	1200	173.5 (34.3)	58	188.0 (44.4)	818	176.8 (45.1)	264	174.0 (44.7)	209	187.1 (50.8)	63
Systolic Blood Pressure [mmHg]	124.0 (17.8)	116	129.0 (21.4)	10	125.5 (18.8)	28	131.2 (15.8)	4	123.5 (18.2)	33	130.5 (21.1)	14	124.2 (16.8)	6	124.1 (18.7)	0
VAT/SAT ratio	.66 (.9)	0	.88 (.8)	0	.73 (2.7)	0	.79 (.5)	0	.63 (.6)	0	.87 (.7)	0	.63 (.6)	0	.80 (.5)	0
Muscle density [HU]	43.9 (9.6)	0	38.2 (9.1)	0	43.7 (9.6)	0	37.7 (7.9)	0	44.0 (9.1)	0	38.3 (8.9)	0	44.2 (9.6)	0	38.9 (8.6)	0

Supplemental Table 4. Imaging scanners and protocol details.

Manufacturer	Scanner Model	Number of Scans	X-ray Tube Current mean (std) in mA	Tube Voltage mode (range) in kV	Reconstruction Kernel (n)	Slice Thickness (mm)
GE MEDICAL SYSTEMS	LightSpeed VCT	2456	460 (187)	120 (80-140)	STANDARD (2453), SOFT (3)	1.25
	LightSpeed16	952	284 (117)	120 (100-140)	STANDARD (952)	1.25
	Discovery CT750 HD	567	331 (143)	100 (80-120)	SOFT (532), STANDARD (35)	1.25
	Revolution CT	510	385 (143)	100 (80-140)	STANDARD (510)	1.25
	LightSpeed Ultra	124	435 (17)	120 (120-140)	SOFT (124)	1.25
	Discovery 690	16	394 (126)	120 (100-120)	STANDARD (9), SOFT (7)	1.25
	Discovery 600	12	308 (122)	120 (120-120)	STANDARD (10), SOFT (2)	1.25
	Discovery 710	6	388 (82)	100 (100-100)	STANDARD (6)	1.25
SIEMENS	SOMATOM Definition Flash	1693	469 (189)	100 (70-140)	I26f (1671), I26s (11), B20f (7), I26f (2), I26s (1), Q30f (1)	1
	SOMATOM Definition Edge	557	453 (113)	100 (80-140)	I26f (556), I26s (1)	1
	SOMATOM Definition AS+	540	441 (119)	100 (80-140)	I26f (344), B20f (86), B31f (64), B31s (16), I26s (14), B20s (9), B25f (5), B18f (1), B30f (1)	1
	Sensation 64	403	360 (97)	120 (80-140)	B20f (283), B31f (118), B31s (1)	1
	SOMATOM Force	245	637 (231)	90 (70-120)	Bf32d (238), Bf32d (3), Bf32s (3), Bv36s (1)	1
	SOMATOM Definition	58	267 (129)	120 (100-120)	B31f (53), B18f (5)	1

Supplemental Table 5. Performance of proposed classification models using Youden's index as threshold, compared to two clinically relevant thresholds for pooled cohort equations (PCE).

Model	1-year IHD risk				5-year IHD risk			
	Sensitivity [%]	Specificity [%]	PPV [%]	NPV [%]	Sensitivity [%]	Specificity [%]	PPV [%]	NPV [%]
PCE (>7.5% threshold)	70.4	71.1	10.1	98.1	61.2	70.5	10.1	84.8
PCE (>20% threshold)	38.0	86.9	11.8	96.8	29.4	86.6	11.8	79.0
Segmentation only	80.3	52.0	7.2	98.3	69.4	62.8	37.8	86.3
Clinical Only	56.3	83.0	13.3	97.6	90.6	48.7	36.5	94.1
Imaging Only	80.3	59.5	8.4	98.5	64.7	77.8	48.7	87.1
Imaging + Clinical Fusion	45.1	87.5	14.2	97.2	69.4	75.5	48.0	88.3

Supplemental Table 6. Performance of proposed prediction models, along with Framingham Risk Score (FRS) and Pooled Cohort Equations (PCE) in different subpopulations of the test set. y/o: years old. (See attached file).
