# Supplementary material for: Opportunistic assessment of ischemic heart disease risk using abdominopelvic computed tomography and medical record data: a multimodal explainable artificial intelligence approach
Source: Sci Rep. 2023 Nov 29;13:21034. doi: 10.1038/s41598-023-47895-y (PMC10687235; doi:10.1038/s41598-023-47895-y)
Supplement: Supplementary file 3 — Supplementary Table 6. [file 41598_2023_47895_MOESM3_ESM.pdf]

Subpopulation	Model	1-year cohort		5-year cohort	
		AUROC (95% CI)	AUCPR (95% CI)	AUROC (95% CI)	AUCPR (95% CI)
Complete PCE data (1y n=328, % IHD positive=3.7) / (5y n=69, % IHD positive=30.4)	FRS	0.64 (0.49-0.77)	0.06 (0.04-0.11)	0.72 (0.61-0.82)	0.47 (0.38-0.64)
	PCE	0.70 (0.57-0.82)	**0.10 (0.06-0.27)**	0.75 (0.65-0.84)	0.53 (0.43-0.72)
	Segmentation only	0.53 (0.39-0.65)	0.04 (0.03-0.07)	0.63 (0.50-0.73)	0.44 (0.34-0.59)
	PCE+Segmentation	0.66 (0.53-0.79)	0.06 (0.05-0.11)	0.69 (0.57-0.80)	0.51 (0.40-0.66)
	Clinical only	0.69 (0.55-0.81)	0.08 (0.05-0.13)	0.81 (0.72-0.90)	0.65 (0.53-0.80)
	Imaging only	0.63 (0.51-0.74)	0.05 (0.04-0.09)	0.74 (0.61-0.84)	0.58 (0.46-0.77)
	Imaging+Clinical Fusion	0.69 (0.57-0.80)	0.07 (0.05-0.14)	0.80 (0.69-0.88)	0.69 (0.57-0.82)
	Imaging+Clinical+Segmentation Fusion	**0.71 (0.60-0.81)**	0.08 (0.06-0.15)	**0.82 (0.72-0.90)**	**0.73 (0.60-0.84)**
Missing PCE data (1y n=1281, % IHD positive=4.6) / (5y n=277, % IHD positive=23.1)	FRS	0.76 (0.71-0.81)	0.11 (0.09-0.15)	0.69 (0.64-0.75)	0.42 (0.35-0.52)
	PCE	0.78 (0.73-0.83)	0.15 (0.12-0.22)	0.73 (0.67-0.78)	0.42 (0.36-0.52)
	Segmentation	0.74 (0.69-0.79)	0.10 (0.08-0.14)	0.68 (0.63-0.75)	0.35 (0.31-0.42)
	PCE+Segmentation	0.79 (0.74-0.83)	0.13 (0.11-0.17)	0.73 (0.67-0.78)	0.39 (0.34-0.48)
	Clinical only	0.82 (0.78-0.86)	0.18 (0.14-0.26)	0.75 (0.70-0.80)	0.47 (0.40-0.57)
	Imaging only	0.79 (0.74-0.83)	0.17 (0.13-0.25)	0.78 (0.72-0.83)	0.55 (0.46-0.65)
	Imaging+Clinical Fusion	**0.84 (0.80-0.87)**	**0.20 (0.15-0.28)**	**0.80 (0.74-0.85)**	0.57 (0.49-0.67)
	Imaging+Clinical+Segmentation Fusion	0.82 (0.77-0.85)	0.19 (0.15-0.28)	**0.80 (0.75-0.85)**	**0.61 (0.52-0.69)**
40-75 y/o (1y n=1051, % IHD positive=4.4)/ (5y n=230, % IHD positive=27.8)	FRS	0.67 (0.61-0.72)	0.07 (0.06-0.09)	0.61 (0.55-0.69)	0.39 (0.33-0.48)
	PCE	0.71 (0.66-0.76)	0.08 (0.07-0.10)	0.64 (0.57-0.71)	0.38 (0.33-0.46)
	Segmentation	0.63 (0.57-0.70)	0.06 (0.05-0.09)	0.61 (0.54-0.68)	0.35 (0.30-0.43)
	PCE+Segmentation	0.71 (0.65-0.76)	0.09 (0.07-0.12)	0.63 (0.57-0.70)	0.35 (0.31-0.42)
	Clinical only	**0.78 (0.72-0.83)**	0.14 (0.10-0.21)	0.68 (0.62-0.74)	0.47 (0.40-0.56)
	Imaging only	0.71 (0.63-0.77)	0.11 (0.08-0.17)	0.73 (0.67-0.79)	0.52 (0.44-0.62)
	Imaging+Clinical Fusion	**0.78 (0.73-0.83)**	0.14 (0.11-0.22)	0.75 (0.69-0.81)	0.56 (0.47-0.66)
	Imaging+Clinical+Segmentation Fusion	0.77 (0.71-0.82)	**0.15 (0.11-0.23)**	**0.76 (0.69-0.81)**	**0.59 (0.51-0.68)**
Under 40 y/o (1y n=433, % IHD positive=1.2) / (5y n=80, % IHD positive=3.8)	FRS	0.50 (0.22-0.80)	0.01 (0.01-0.03)	0.42 (0.01-0.38)	0.07 (0.03-0.24)
	PCE	0.49 (0.12-0.80)	0.02 (0.01-0.07)	0.42 (0.04-0.25)	0.14 (0.03-0.60)
	Segmentation	0.62 (0.28-0.92)	0.04 (0.01-0.16)	0.54 (0.25-0.53)	0.06 (0.04-0.16)
	PCE+Segmentation	0.65 (0.50-0.91)	0.05 (0.02-0.28)	0.41 (0.28-0.57)	0.04 (0.04-0.06)
	Clinical only	0.76 (0.47-0.95)	0.06 (0.02-0.15)	0.67 (0.34-0.72)	0.11 (0.05-0.38)
	Imaging only	0.79 (0.52-0.95)	0.05 (0.03-0.13)	0.73 (0.36-0.99)	0.17 (0.06-0.52)
	Imaging+Clinical Fusion	**0.80 (0.50-0.96)**	**0.06 (0.03-0.14)**	0.76 (0.33-0.97)	0.19 (0.07-0.57)

	Imaging+Clinical+Segmentation Fusion	**0.80 (0.51-0.94)**	**0.06 (0.03-0.12)**	**0.79 (0.41-0.99)**	**0.22 (0.08-0.69)**
Over 75 y/o (1y n=125, % IHD positive=16.0) / (5y n=36, % IHD positive=50.0)	FRS	0.52 (0.42-0.64)	0.16 (0.14-0.22)	0.49 (0.35-0.69)	0.62 (0.50-0.76)
	PCE	**0.58 (0.45-0.70)**	**0.21 (0.17-0.33)**	0.57 (0.44-0.75)	0.60 (0.49-0.78)
	Segmentation	0.54 (0.42-0.67)	0.20 (0.15-0.32)	0.44 (0.26-0.59)	0.47 (0.41-0.63)
	PCE+Segmentation	0.53 (0.44-0.64)	0.18 (0.15-0.23)	0.63 (0.46-0.79)	0.72 (0.60-0.84)
	Clinical only	0.55 (0.43-0.67)	0.20 (0.16-0.33)	0.67 (0.49-0.80)	0.69 (0.56-0.84)
	Imaging only	0.52 (0.40-0.60)	0.19 (0.15-0.31)	**0.81 (0.65-0.92)**	**0.85 (0.76-0.95)**
	Imaging+Clinical Fusion	**0.58 (0.45-0.67)**	0.20 (0.16-0.32)	0.78 (0.61-0.91)	0.80 (0.67-0.94)
	Imaging+Clinical+Segmentation Fusion	0.56 (0.42-0.65)	0.20 (0.16-0.31)	**0.81 (0.66-0.92)**	**0.85 (0.76-0.94)**
Male (1y n=668, % IHD positive=5.8) / (5y n=128, % IHD positive=27.3)	FRS	0.71 (0.63-0.77)	0.13 (0.10-0.20)	0.67 (0.59-0.76)	0.43 (0.35-0.54)
	PCE	0.70 (0.63-0.77)	0.14 (0.10-0.22)	0.70 (0.62-0.78)	0.49 (0.39-0.60)
	Segmentation	0.68 (0.60-0.74)	0.11 (0.08-0.17)	0.63 (0.56-0.73)	0.36 (0.31-0.48)
	PCE+Segmentation	0.71 (0.63-0.77)	0.12 (0.09-0.19)	0.70 (0.62-0.79)	0.44 (0.36-0.58)
	Clinical only	0.76 (0.69-0.82)	0.18 (0.13-0.29)	0.70 (0.62-0.78)	0.51 (0.41-0.63)
	Imaging only	0.74 (0.67-0.79)	0.13 (0.11-0.20)	0.83 (0.75-0.89)	0.71 (0.61-0.81)
	Imaging+Clinical Fusion	**0.78 (0.71-0.83)**	0.18 (0.13-0.28)	**0.83 (0.76-0.89)**	0.70 (0.59-0.81)
	Imaging+Clinical+Segmentation Fusion	0.76 (0.70-0.82)	**0.19 (0.13-0.29)**	**0.83 (0.76-0.90)**	**0.72 (0.62-0.83)**
Female (1y n=941, % IHD positive=3.4) / (5y n=218, % IHD positive=22.9)	FRS	0.82 (0.77-0.86)	0.10 (0.08-0.14)	0.75 (0.70-0.82)	0.47 (0.39-0.57)
	PCE	0.83 (0.78-0.87)	0.14 (0.10-0.23)	0.75 (0.71-0.82)	0.44 (0.37-0.56)
	Segmentation	0.76 (0.71-0.82)	0.07 (0.06-0.12)	0.71 (0.65-0.78)	0.37 (0.32-0.47)
	PCE+Segmentation	0.81 (0.75-0.85)	0.11 (0.08-0.15)	0.74 (0.68-0.80)	0.41 (0.34-0.52)
	Clinical only	0.84 (0.79-0.88)	0.13 (0.10-0.20)	0.79 (0.75-0.85)	0.52 (0.43-0.64)
	Imaging only	0.77 (0.69-0.84)	**0.15 (0.09-0.24)**	0.74 (0.68-0.81)	0.48 (0.40-0.59)
	Imaging+Clinical Fusion	**0.84 (0.79-0.88)**	0.13 (0.10-0.21)	0.78 (0.72-0.84)	0.53 (0.44-0.64)
	Imaging+Clinical+Segmentation Fusion	0.82 (0.76-0.87)	0.13 (0.10-0.20)	**0.79 (0.73-0.85)**	**0.58 (0.48-0.67)**
Asian (1y n=255, % IHD positive=5.1) / (5y n=50, % IHD positive=32.0)	FRS	0.81 (0.72-0.89)	0.16 (0.11-0.27)	0.76 (0.67-0.90)	0.67 (0.53-0.83)
	PCE	**0.85 (0.75-0.92)**	0.25 (0.16-0.46)	0.78 (0.68-0.89)	0.61 (0.49-0.77)
	Segmentation	0.80 (0.71-0.88)	0.22 (0.11-0.39)	0.74 (0.66-0.89)	0.58 (0.45-0.80)
	PCE+Segmentation	0.78 (0.67-0.87)	0.15 (0.10-0.23)	0.75 (0.64-0.88)	0.56 (0.43-0.74)
	Clinical only	0.83 (0.73-0.92)	0.30 (0.16-0.51)	0.84 (0.77-0.94)	0.71 (0.57-0.88)
	Imaging only	0.77 (0.65-0.86)	0.23 (0.11-0.43)	0.88 (0.79-0.95)	0.73 (0.59-0.93)
	Imaging+Clinical Fusion	**0.85 (0.75-0.93)**	**0.33 (0.18-0.55)**	0.89 (0.81-0.96)	0.83 (0.71-0.94)
	Imaging+Clinical+Segmentation Fusion	0.83 (0.72-0.92)	0.31 (0.17-0.52)	**0.90 (0.82-0.97)**	**0.86 (0.76-0.95)**

Non-Hispanic Black (1y n=50, % IHD positive=4.0)/ (5y n=17, % IHD positive=17.6)	FRS	**0.94 (0.84-1.00)**	0.39 (0.22-1.00)	**1.00 (1.00-1.00)**	**1.00 (1.00-1.00)**
	PCE	0.83 (0.69-0.96)	0.19 (0.11-0.67)	0.90 (0.77-1.00)	0.70 (0.42-1.00)
	Segmentation	0.79 (0.57-0.98)	0.22 (0.09-0.67)	0.90 (0.69-1.00)	0.70 (0.42-1.00)
	PCE+Segmentation	0.89 (0.78-0.98)	0.22 (0.14-0.67)	0.94 (0.73-1.00)	0.83 (0.58-1.00)
	Clinical only	0.88 (0.79-0.95)	0.19 (0.14-0.50)	0.90 (0.69-1.00)	0.72 (0.44-1.00)
	Imaging only	0.80 (0.53-1.00)	**0.55 (0.08-1.00)**	0.69 (0.19-0.88)	0.54 (0.22-0.92)
	Imaging+Clinical Fusion	0.90 (0.76-1.00)	0.34 (0.15-1.00)	0.81 (0.47-0.94)	0.61 (0.31-1.00)
	Imaging+Clinical+Segmentation Fusion	0.86 (0.69-0.98)	0.24 (0.13-0.67)	0.86 (0.47-1.00)	0.78 (0.49-1.00)
Hispanic (1y n=328, % IHD positive=4.9)/ (5y n=65, % IHD positive=21.5)	FRS	0.74 (0.61-0.86)	0.16 (0.10-0.30)	0.73 (0.58-0.87)	0.53 (0.37-0.72)
	PCE	0.75 (0.59-0.86)	0.22 (0.13-0.43)	0.78 (0.65-0.91)	0.58 (0.42-0.76)
	Segmentation	0.72 (0.61-0.82)	0.12 (0.08-0.26)	0.67 (0.58-0.83)	0.39 (0.27-0.59)
	PCE+Segmentation	0.79 (0.67-0.86)	0.14 (0.10-0.23)	0.72 (0.60-0.89)	0.43 (0.32-0.66)
	Clinical only	0.80 (0.69-0.86)	0.16 (0.11-0.30)	0.76 (0.67-0.88)	0.48 (0.34-0.67)
	Imaging only	0.79 (0.67-0.89)	0.21 (0.12-0.36)	0.83 (0.72-0.92)	**0.62 (0.46-0.79)**
	Imaging+Clinical Fusion	**0.81 (0.69-0.88)**	0.20 (0.12-0.36)	0.85 (0.76-0.94)	**0.62 (0.47-0.81)**
	Imaging+Clinical+Segmentation Fusion	0.80 (0.70-0.87)	**0.21 (0.12-0.38)**	**0.86 (0.77-0.94)**	**0.62 (0.47-0.81)**
Other (1y n=170, % IHD positive=4.7) / (5y n=36, % IHD positive=30.6)	FRS	0.66 (0.49-0.75)	0.08 (0.06-0.14)	0.69 (0.52-0.82)	0.43 (0.35-0.67)
	PCE	0.78 (0.65-0.85)	0.12 (0.09-0.23)	0.75 (0.60-0.88)	0.46 (0.37-0.70)
	Segmentation	0.76 (0.58-0.85)	0.14 (0.09-0.33)	0.75 (0.61-0.87)	0.46 (0.37-0.70)
	PCE+Segmentation	0.77 (0.65-0.89)	0.12 (0.08-0.22)	0.77 (0.63-0.89)	0.48 (0.39-0.71)
	Clinical only	0.79 (0.67-0.91)	0.14 (0.10-0.35)	0.76 (0.61-0.88)	0.58 (0.44-0.77)
	Imaging only	**0.87 (0.83-0.91)**	0.16 (0.14-0.25)	0.78 (0.68-0.92)	0.55 (0.42-0.80)
	Imaging+Clinical Fusion	0.83 (0.75-0.92)	**0.17 (0.11-0.39)**	**0.81 (0.69-0.92)**	0.58 (0.44-0.79)
	Imaging+Clinical+Segmentation Fusion	0.81 (0.75-0.92)	0.16 (0.10-0.38)	0.80 (0.69-0.92)	**0.59 (0.45-0.81)**
Non-Hispanic White (1y n=806, % IHD positive=4.0) / (5y n=178, % IHD positive=23.0)	FRS	0.71 (0.63-0.77)	0.07 (0.06-0.10)	0.67 (0.61-0.75)	0.36 (0.29-0.46)
	PCE	0.73 (0.66-0.79)	0.08 (0.07-0.14)	0.68 (0.62-0.77)	0.40 (0.32-0.50)
	Segmentation	0.64 (0.57-0.71)	0.06 (0.05-0.07)	0.64 (0.56-0.72)	0.32 (0.27-0.42)
	PCE+Segmentation	0.74 (0.67-0.81)	0.10 (0.07-0.15)	0.67 (0.60-0.75)	0.37 (0.30-0.50)
	Clinical only	**0.79 (0.73-0.85)**	0.13 (0.09-0.20)	0.72 (0.66-0.80)	0.45 (0.36-0.57)
	Imaging only	0.71 (0.63-0.78)	0.09 (0.07-0.14)	0.73 (0.65-0.81)	0.55 (0.44-0.68)
	Imaging+Clinical Fusion	**0.79 (0.74-0.85)**	0.12 (0.09-0.19)	0.75 (0.67-0.83)	0.57 (0.46-0.69)
	Imaging+Clinical+Segmentation Fusion	**0.79 (0.72-0.85)**	**0.14 (0.10-0.22)**	**0.76 (0.68-0.84)**	**0.60 (0.50-0.71)**
	FRS	0.74 (0.66-0.79)	0.10 (0.08-0.15)	0.71 (0.65-0.79)	0.45 (0.37-0.57)

Taking lipid modifying agents (1y n=809, % IHD positive=4.6) / (5y n=173, % IHD positive=24.9)	PCE	0.77 (0.71-0.83)	0.13 (0.10-0.22)	0.71 (0.66-0.79)	0.42 (0.34-0.54)
	Segmentation	0.71 (0.65-0.78)	0.09 (0.07-0.12)	0.69 (0.61-0.76)	0.40 (0.33-0.52)
	PCE+Segmentation	0.76 (0.69-0.81)	0.11 (0.09-0.15)	0.70 (0.64-0.77)	0.39 (0.33-0.50)
	Clinical only	**0.81 (0.77-0.87)**	**0.15 (0.11-0.22)**	0.76 (0.71-0.83)	0.53 (0.44-0.64)
	Imaging only	0.73 (0.66-0.79)	0.10 (0.08-0.17)	0.74 (0.66-0.81)	0.58 (0.48-0.69)
	Imaging+Clinical Fusion	0.80 (0.75-0.86)	0.14 (0.11-0.23)	**0.77 (0.70-0.84)**	0.61 (0.52-0.71)
	Imaging+Clinical+Segmentation Fusion	0.79 (0.73-0.85)	0.14 (0.11-0.22)	0.77 (0.71-0.84)	**0.62 (0.53-0.72)**
Not taking lipid modifying agents (1y n=800, % IHD positive=4.2) / (5y n=173, % IHD positive=24.3)	FRS	0.73 (0.66-0.80)	0.10 (0.08-0.14)	0.70 (0.63-0.78)	0.43 (0.35-0.54)
	PCE	0.76 (0.68-0.82)	0.14 (0.10-0.23)	0.76 (0.69-0.82)	0.50 (0.40-0.61)
	Segmentation	0.69 (0.61-0.76)	0.08 (0.06-0.14)	0.67 (0.61-0.76)	0.35 (0.30-0.45)
	PCE+Segmentation	0.77 (0.70-0.83)	0.11 (0.09-0.16)	0.74 (0.68-0.82)	0.47 (0.38-0.58)
	Clinical only	0.79 (0.72-0.84)	0.17 (0.11-0.27)	0.76 (0.70-0.83)	0.52 (0.43-0.64)
	Imaging only	0.80 (0.73-0.86)	0.16 (0.12-0.26)	0.81 (0.75-0.87)	0.57 (0.47-0.70)
	Imaging+Clinical Fusion	**0.82 (0.76-0.87)**	0.19 (0.13-0.32)	**0.84 (0.77-0.89)**	0.60 (0.51-0.75)
	Imaging+Clinical+Segmentation Fusion	0.81 (0.75-0.86)	**0.21 (0.13-0.33)**	**0.84 (0.78-0.89)**	**0.66 (0.56-0.78)**
Acute IHD (I21,I22) positives only (1y n=1552, % IHD positive=0.9) / (5y n=281, % IHD positive=7.1)	FRS	0.71 (0.55-0.83)	0.02 (0.02-0.04)	0.72 (0.62-0.82)	0.19 (0.11-0.30)
	PCE	0.76 (0.60-0.88)	0.03 (0.02-0.07)	0.72 (0.60-0.80)	0.18 (0.12-0.31)
	Segmentation	0.70 (0.61-0.81)	0.02 (0.01-0.03)	0.73 (0.63-0.80)	0.15 (0.11-0.25)
	PCE+Segmentation	0.78 (0.64-0.89)	0.03 (0.02-0.05)	0.73 (0.63-0.81)	0.18 (0.12-0.31)
	Clinical only	0.79 (0.69-0.89)	0.03 (0.02-0.07)	0.75 (0.65-0.83)	0.31 (0.18-0.49)
	Imaging only	**0.81 (0.69-0.90)**	0.03 (0.02-0.06)	0.86 (0.77-0.92)	0.44 (0.29-0.62)
	Imaging+Clinical Fusion	**0.81 (0.68-0.91)**	**0.04 (0.03-0.08)**	**0.87 (0.78-0.93)**	0.46 (0.32-0.63)
	Imaging+Clinical+Segmentation Fusion	**0.81 (0.70-0.91)**	**0.04 (0.03-0.08)**	0.86 (0.77-0.93)	**0.49 (0.34-0.67)**
non-acute IHD (I20,I23,I24,I25) positives only (1y n=1595, % IHD positive=3.6) / (5y n=326, % IHD positive=19.9)	FRS	0.74 (0.70-0.79)	0.08 (0.07-0.11)	0.70 (0.65-0.76)	0.38 (0.31-0.48)
	PCE	0.77 (0.72-0.81)	0.11 (0.08-0.17)	0.74 (0.69-0.79)	0.38 (0.32-0.48)
	Segmentation	0.70 (0.65-0.75)	0.07 (0.06-0.10)	0.67 (0.61-0.73)	0.29 (0.25-0.36)
	PCE+Segmentation	0.76 (0.72-0.80)	0.09 (0.07-0.11)	0.72 (0.66-0.77)	0.34 (0.29-0.43)
	Clinical only	0.80 (0.76-0.84)	0.12 (0.10-0.18)	0.76 (0.71-0.81)	0.44 (0.37-0.54)
	Imaging only	0.75 (0.70-0.80)	0.10 (0.08-0.16)	0.75 (0.69-0.80)	0.45 (0.37-0.55)
	Imaging+Clinical Fusion	**0.81 (0.77-0.85)**	**0.13 (0.10-0.19)**	0.78 (0.73-0.83)	0.50 (0.42-0.60)
	Imaging+Clinical+Segmentation Fusion	0.80 (0.75-0.83)	**0.13 (0.10-0.19)**	**0.79 (0.73-0.84)**	**0.55 (0.47-0.64)**
